# WPSAR after 14 volumes: achievements and future directions

**DOI:** 10.5365/wpsar.2023.14.4.1136

**Published:** 2023-12-31

**Authors:** Ashley Arashiro, Michelle McPherson, Roxanne Andaya, Don Rivada, Babatunde Olowokure

**Affiliations:** a WPSAR Editorial Team, WHO Health Emergencies Programme, World Health Organization Regional Office for the Western Pacific, Manila, Philippines.

Since 2010, the *Western Pacific Surveillance and Response* (WPSAR) journal has served as a platform for timely information-sharing on the surveillance of and response to public health events and emergencies in the Western Pacific Region. WPSAR publishes a broad range of articles not limited to conventional research articles and, unlike many other scientific journals, builds capacity in communicating epidemiological and operational findings by providing pre-submission assistance to first-time authors in the Region, particularly those who are fellows or recent graduates of field epidemiology training programmes (FETPs).

WPSAR is the official journal of the World Health Organization (WHO) Regional Office for the Western Pacific and the scientific communication component of the Region’s efforts against health security threats. The journal welcomes contributions from field epidemiologists, staff of Ministries and Departments of Health and other government officials, those working in health security and global health, other front-line professionals responding to public health events and emergencies in the Region, and specialists from other disciplines and settings such as academia and clinical practice.

One of the most widely debated journal metrics is impact factor. The impact factor of a journal, as calculated by Clarivate, is a measure of the average number of citations that articles published in the two prior years received in the current year. (*1*) In June 2023, WPSAR received its first impact factor of 1.0. This means that every article published in WPSAR in 2020 and 2021 was cited by another scientific article an average of 1.0 time in 2022. This is an exciting milestone in the journal’s history, as it serves as recognition of the editorial team’s efforts to consistently publish timely, high-quality articles. In this editorial, we mark this achievement with a summary of the journal’s work over the course of 14 volumes and a look at future directions for WPSAR.

Over the past 14 years, WPSAR has consistently published four quarterly issues per year, plus special editions on timely topics. These special editions have covered the response to Typhoon Haiyan in the Philippines (2015), (*2*) the centennial of the 1918 influenza pandemic (2018), (*3*) COVID-19 clinical management and health-care pathways (2023) (*4*) and the establishment of emergency medical teams in the Pacific (2023). (*5*) Additionally, while COVID-19 was declared a public health emergency of international concern by WHO (January 2020 to May 2023), (*6*) each quarterly issue featured a section dedicated to COVID-19 articles.

**Fig. 1:**
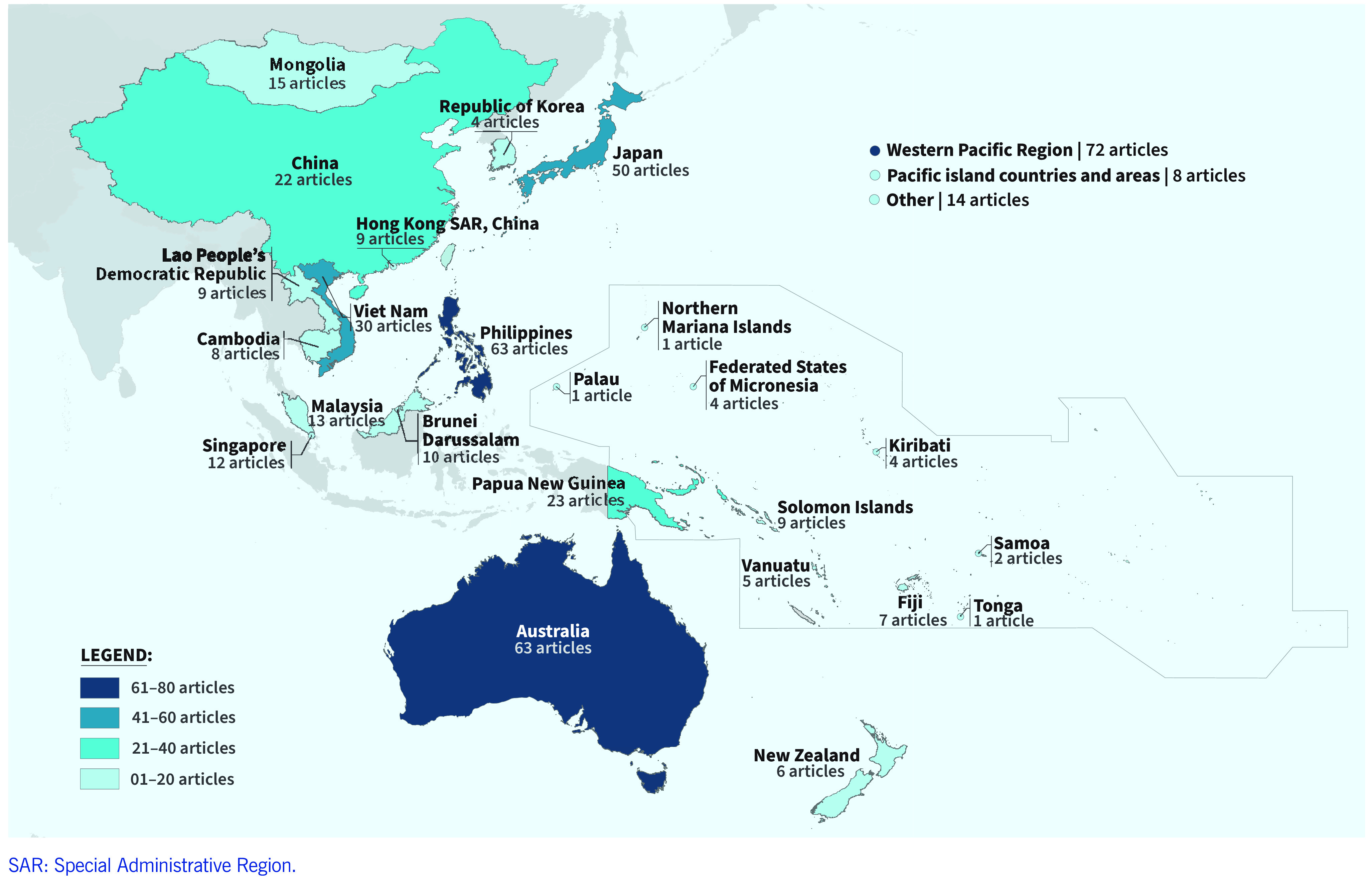
Published articles in WPSAR by country and area (N = 465)

As of December 2023, WPSAR has cumulatively published 465 articles, 179 (38.5%) of them in the past 5 years. The most frequently published article type is Original Research, followed by Outbreak Investigation Report, Brief Report and Surveillance Report (**Table 1**). By country and area, the highest number of articles cover Region-wide topics (*n* = 72), followed by those from Australia and the Philippines (*n* = 63 each), Japan (*n* = 50), Viet Nam (*n* = 30), Papua New Guinea (*n* = 23) and China (*n* = 22, excluding Hong Kong Special Administrative Region SAR, China) (**Fig. 1**). The acceptance rate of articles that met the scope of the journal was 87% (47/54) in 2021 and 83% (45/54) in 2022. While showing a slight decrease, this figure is high due to the policy of accepting all submissions that meet the scope of the journal and offering pre-submission assistance to authors to ensure their articles meet publication standards, in line with the objective of building scientific writing capacity in the Region.

[**insert** Figure 1]

**Table 1:** Published articles in WPSAR by article type (*n* = 465)

Article type	*n*(%)
Original Research	137 (29.5)
Outbreak Investigation Report	54 (11.6)
Brief Report	48 (10.3)
Surveillance Report	46 (9.9)
Lessons from the Field	41 (8.8)
Perspective	38 (8.2)
Regional Analysis	26 (5.6)
Field Investigation	19 (4.1)
Editorial	15 (3.2)
Surveillance System Implementation/Evaluation	13 (2.8)
Case Report/Case Series	11 (2.4)
Letter to the Editor	8 (1.7)
Risk Assessment	7 (1.5)
News, Meeting and Conference Report	1 (0.2)
Miscellaneous	1 (0.2)

In recent years, WPSAR has expanded its global team of associate editors to offer a wider diversity of expertise during article review. Several initiatives are also underway to reduce the time from article submission to publication, such as expanding the pool of external independent peer reviewers and implementing technology and process improvements. The Executive Editor and the editorial team may select certain manuscripts that are of urgent public health importance for expedited early publication.

In October 2023, the draft *Asia Pacific Health Security Action Framework* was submitted to the 74th session of the WHO Regional Committee Meeting for the Western Pacific, where it was endorsed by Member States. (*7*) This Framework is the successor to the *Asia Pacific Strategy for Emerging Diseases and Public Health Emergencies* (APSED III) (*8*) and will be implemented by Member States beginning in 2024. Building on the experiences from its previous iterations, the Framework identifies six multisectoral domains that interconnect to form a comprehensive health security system that can be implemented flexibly at subnational, national and regional levels. (*9*)

In support of the new Framework, the WPSAR editorial team announces the expansion of the journal’s scope to include all aspects of health security in response to public health events and emergencies. WPSAR will especially aim to address all activities related to the prevention, preparedness, readiness, response and recovery to public health events and emergencies, prioritizing topics that are of relevance to the Western Pacific Region. Public health events may be acute or ongoing, and topics to be explored can fall under any of the following areas: communicable diseases, emerging infectious diseases, natural disasters, food safety, bioterrorism, and chemical and radiological events. Other events and topics may also be considered as the journal seeks to disseminate important insights that can lead to improved protection of people’s health before, during and after outbreaks, epidemics, pandemics, disasters and other public health emergencies.

The WPSAR editorial team would like to thank our authors, anonymous reviewers and associate editors for their outstanding contributions to our success over the past 14 years. We look forward to continuing to work with you. We also look forward to welcoming articles within the expanded scope of the journal and continuing to support and promote the work of all those involved in health security across the Region.
